# Effects of Variety and Postharvest Handling Practices on Microbial Population at Different Stages of the Value Chain of Fresh Tomato* (Solanum lycopersicum)* in Western Terai of Nepal

**DOI:** 10.1155/2017/7148076

**Published:** 2017-10-12

**Authors:** Ram B. Khadka, Madan Marasini, Ranjana Rawal, Durga M. Gautam, Antonio L. Acedo

**Affiliations:** ^1^Regional Agricultural Research Station, Nepalgunj, Banke, Nepal; ^2^Agriculture and Forestry University, Rampur, Chitwan, Nepal; ^3^World Vegetable Center South Asia, Hyderabad, India

## Abstract

*Background*. Fresh vegetables such as tomato should have low microbial population for safe consumption and long storage life. The aerobic bacterial count (ABC) and coliform bacterial count (CBC), yeast, and mold population are the most widely used microbial indicators in fresh vegetables which should be lower than 4 log CFU g^−1^ for safe consumption. The stages of the supply chain, postharvest handling methods, and crop varieties had significant effects on microbial population. ABC, CBC, yeast, and mold population were significantly highest (*P* < 0.05) at retail market (5.59, 4.38, 2.60, and 3.14 log CFU g^−1^, resp.), followed by wholesale market (4.72, 4.71, 2.43, and 2.44 log CFU g^−1^, resp.), and were least at farm gate (3.89, 3.63, 2.38, and 2.03 log CFU g^−1^, resp.). Improved postharvest practices (washing in clean water and grading and packaging in clean plastic crate) helped to reduce ABC, CBC, and mold population by 2.51, 32.70, and 29.86 percentage as compared to the conventional method (no washing and no grading and packaging in mud plastered bamboo baskets). Among varieties, Pusa ruby had the lowest microbial load of 2.58, 4.53, 0.96, and 1.77 log CFU g^−1^ for ABC, CBC, yeast, and mold count, respectively. Significantly negative correlation (*P* < 0.05) was observed between fruit pH & ABC and pH & mold count. Although the microbial quality of fresh tomato is safe in the local market of western Terai of Nepal both in conventional and in improved practices however still it is essential to follow improved postharvest handling practices in production and marketing of newly introduced tomato cultivars (high-pH cultivars) for ensuring the safe availability of fresh tomato in the market.

## 1. Introduction

Tomato (*Solanum lycopersicum* L.) is one of the most important perishable vegetables of Nepal. It is projected that 25 to 50% of the total production of tomato is lost at the postproduction stage [1, 2]. Therefore, to reduce production loss, the postharvest handling and management are the crucial work in tomato production. The postharvest loss is mostly associated with microbial contamination which fosters spoilage during transportation, storage, and marketing. These microbes may contaminate tomato fruit at both preharvest and postharvest stages. The preharvest sources of microbial contaminants are soil, fertilizer, compost, irrigation water, and pesticide solution while postharvest sources include soil, cleaning and treating waters, packing shed, transporting equipment, and storage [3, 4]. Generally, the contamination occurred due to poor production and handling practices such as the application of contaminated water in irrigation and cleaning up and unhygienic handling practices. These contaminated microbes can produce toxin and secondary metabolites, which may cause the serious health issues [5]. Therefore these are included in the group of health hazard [6]. The microbial load of the perishable goods is the main criteria to determine the shelf life of the perishable agricultural products [7]. These microbes include Gram-negative bacteria, Gram-positive bacteria, fungi (yeasts and molds), viruses, and parasites [8].

Coliforms are facultative anaerobic, Gram-negative, non-spore-forming rods shaped bacteria including the heterogeneous groups. Coliforms are common habitant of mammalian guts [8].* E. coli* is the common species of coliform group mostly associated with fecal contamination. Therefore, coliforms bacteria are used to evaluate the general hygiene level and are the best indicator of fecal pollution. The aerobic bacterial (AB) population is one of the most important microbiological indicators for food quality. These bacterial loads reflect the exposure of the sample to any contamination, and, in general, this is the indicator of sanitation level maintained during transportation, handling, and storage [9].

Understanding the microbial profile in particular commodity is necessary to reduce microbial population with specific treatments. The present study was conducted to scrutinize the microorganism associated with tomato postharvest life, determine the population load in different stages of supply chain, and evaluate the varietal response with the microbial colonization at western Terai condition of Nepal.

## 2. Materials and Methods

### 2.1. Collection of Samples

A total of 42 samples each having 1 kg of tomato were randomly collected from the traders at three different points of supply chains: farm gate, wholesaler, and retailer during February and March 2016 in Banke district of Nepal. Each 21 samples were representing traditional and improved system of postharvest handling (Table 1). The samples were collected from Hariyali Vegetable Farmer's group, Manakamana of Banke district. All the samples in different stages were collected from the same lot and site of production as much as possible. The samples from wholesale market were collected from Ranitalau Vegetable Collection and Marketing Center, Nepalgunj, Banke, and the samples from retail market were from the local store of Khajura, Banke (owner Bindeshwor Shaha). Seven replicates of samples representing conventional and improved methods were collected from each stage (farm gate, wholesaler, and retailer). The samples were placed in properly labeled sterile polyethylene plastic bags and brought to the laboratory. The samples were stored in a refrigerator at 4°C for 24 hours before microbial analysis [10].

Similarly, 12 samples representing each experimental unit having three replicates of four cultivars were randomly collected from the tomato varietal trial conducted under USAID-AVRDC (United States Agency for International Development-Asian Vegetable Research and Development Center) postharvest project at Regional Agricultural Research Station (RARS), Khajura, Banke, Nepal. The tomato was harvested at pick harvesting (red ripe) stage. Four cultivars—AVTO 1418 (CLN369A), AVTO 1432 (CLN 3940), AVTO 9331 (UC204A) and Pusa ruby—were used in the study. Among them the first 3 were recently introduced from AVRDC, Taiwan, whereas the last one is locally well adapted popular cultivar. Tomato was macerated using mixture grinder to extract juice and extract was filtered by muslin cloth and pH was measured using benchtop pH meter (Thermo-Scientific; Orion 2-Star Benchtop pH Meter).

### 2.2. Methods of Postharvest Handling

Two methods of postharvest handling—conventional and improved practice—were compared in the study. The critical differences between the two methods are presented in Table 1.

### 2.3. Microbial Culture and Enumeration

From one Kg composite tomato samples, 50 g was randomly selected and placed in the sterile high-speed blender jar, added with 450 ml of sterile Butterfield's phosphate-buffered water (34 g KH_2_PO_4_ in 1 L distilled water adjusted pH 7.2 with 1 N NaOH) [10] and blended for 2 min. The homogenate was considered as diluent having dilution factor 10^−1^. The homogenate was further diluted at 10^−3^, 10^−5^, 10^−7^, and 10^−9^ by adding 1 ml in each 90-ml diluent (Butterfield's phosphate-buffered water) using separate sterile pipettes. All dilutions were shaken in vortex for 2-3 minutes. One ml of each dilution was pipetted into separate glass Petri plates having different media. The dilution was shaken each time before pipetting into the Petri plate. Standard agar plate methods were used for microbial enumeration; plate count agar (PCA; 5.0 g tryptone, 2.5 g yeast extract, 1.0 g dextrose, 15.0 g agar, and 1-liter distilled water) was used for total aerobic bacteria count [11, 12]; violet red bile agar (VRBA, 41.5 g of VRBA in 1 liter distilled water; HiMedia Laboratories Pvt. Ltd.) [13] was used for coliform count; and Chloramphenicol Yeast Glucose Agar (CYGA; dextrose, 20.0 g; yeast extract, 5.0 g; chloramphenicol, 0.1 g; agar, 15.0) was used for yeast and mold count [12, 14]. The microbial cultures were incubated for specified duration under ambient conditions (27–33°C).

For the determination of aerobic bacterial count, 20 ml of PCA was plated in 90 mm glass plates, and 1 ml of each decimal dilution was added to each plate, and the plates were incubated at 30 ± 2°C. Then the bacterial colonies were counted and expressed as CFU g^−1^. For determining the coliform bacteria, 1 ml of each dilution was poured in Petri plates, and then 15 ml VRBA medium was added over and circled both clockwise and anticlockwise for good mix and allowed to solidify. Then the plates were incubated in 35 ± 2°C for 24 hours. Finally, red colonies were counted. For determination of yeasts and molds, 1 mL of each decimal dilution was added to plate surface that contained CYGA and distributed by a sterilized L-shaped spreader. The plates were incubated at 25 ± 2°C for five days, and the colonies were counted in each plate and expressed as CFU g^−1^. Each of the plates was replicated two times for each of the dilution factors; however measurement was done in only one plate.

Microbial counts were determined using dilution plates with 15–300 colonies expressed as colony forming units per ml (CFU ml^−1^) [15]. When CFU exceeded 300 per plate, counts were taken from four 1-cm squares per plate. Finally, logarithmic values of counts (log CFU ml^−1^) were computed for every plate.

In addition, pH of tomato fruit of the four different varieties was recorded using a pH meter, three times for each experimental unit.

### 2.4. Data Analysis

The first experiment was conducted in split plot design with seven replications where the methods of postharvest handling were main plot and the stages in supply chain were subplot whereas the second experiment was conducted in randomized complete block design with 3 replications where four cultivars CLN369A, CLN 3940, and UC204A including local check Pusa ruby were evaluated. Both the experiments were conducted twice. The log-transformed value of the colony forming unit per gram was analyzed. Data analysis was done with Microsoft Excel (2016) and R-Studio Version 0.99.896. Data were subjected to analysis of variance (ANOVA); when differences were found, means were separated using Duncan's Multiple Range Test (DMRT).

## 3. Results and Discussions

### 3.1. The Microbial Population in Different Stages of Supply Chain

**Figure 1:**
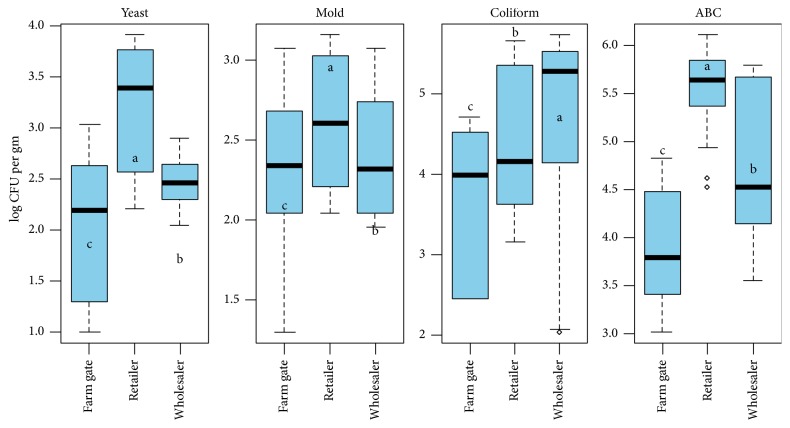
The microbial population in fresh tomato at different stages in supply chain. Means followed by the same letter(s) do not differ significantly at 1% level of probability.

There is a very limited study on the microbiological quality of fresh vegetable in western Terai of Nepal. This study may be the first report from a western part of the country. The log CFU AB, coliform, and mold count at different points of tomato supply chain are presented in Figure 1. The microbial population was found to be significantly changed over the supply chain (*P* < 0.05). The significantly highest (*P* < 0.01) aerobic bacterial count (ABC) was recorded in the retail market (log 5.52 CFU ml^−1^) followed by wholesale (log 4.72) and the lowest one was at farm gate (log 3.89 CFU ml^−1^). Similar results were also recorded in case of coliform, mold, and yeast population. The highest log-transformed CFU of coliform, mold, and yeast were recorded in the retail market (4.38, 2.60, and 3.14, resp.) and the lowest ones were in farm gate (3.89, 3.63, and 2.02) among the three different points of supply chain. The result indicates the microbiological quality of fresh tomato in farm gate can be considered safe as per the HACCP-TQM (Hazard Analysis and Critical Control Points-Total Quality Management) guidelines which were less than 4 log CFU g^−1^. While the aerobic and coliform bacterial counts are not safe at retailer and wholesale market which were higher than 4 log CFU g^−1^, the coliform and aerobic bacteria are the important indicators of hygienic production, transportation, and handling.

This indicates the higher population in retailer and wholesaler may be due to use of waste water in cleaning and improper handling in transportation and use of dirty crates or baskets.

In general, total counts of microbiological populations on minimally processed vegetables after processing range from 3.0 to 6.0 log CFU g^−1^ [16]. Most reported counts for total aerobic bacteria ranged between 4 and 8 log CFU g^−1^ and between 0.7 and 6 log CFU g^−1^ for coliforms. According to the HACCP-TQM technical guidelines, raw foods containing less than 4.0 log CFU g^−1^ (number of spoilage microorganisms, aerobic plate count at 21.1°C) are rated as “good,” with 4.0–6.7 as “average,” 6.7–7.7 as “poor,” and more than 7.7 as “spoiled food,” respectively. Thus, in general the log transferred CFU g^−1^ value of any microbes below 4 is considered as the nondetectable level in this study. Most of the developed countries have their own rules and regulations regarding the minimal microbiological loads in fresh vegetable and fruit to be sold in market. For example, according to the French regulation, the maximum acceptable value of ABC is 5 × 10^7^ CFU g^−1^ [17].

### 3.2. The Microbial Population in Improved and Conventional Postharvest Handling Practice in Fresh Tomato

The method and means of transportation have the major role in fresh vegetable contaminant with hazardous microbes. In this study, basically, the microbial load in two different systems of postharvest management was analyzed. The result showed the postharvest practices have significantly changed the microbial load in fresh tomato in western Terai of Nepal. The result indicates significantly higher (*P* < 0.01, except ABC) log-transformed CFU g^−1^ of aerobic, coliform, and mold count in conventional (4.77, 5.07, and 2.98, resp.) as compared to improved (4.65, 3.41, and 2.09, resp.; Table 2) method, while the mold population was recorded higher in improved practice (2.59 versus 2.35) as compared to conventional. The results indicate the average population load of coliform and aerobic bacteria was found above detectable level in both practices whereas the mold and yeast population were below detectable level in both practices.

The species composition of microbes isolated from the fresh vegetable and fruits at farm gate is the reflector of the microbial profile present in the field at harvesting time [3] while these populations at retailer and wholesaler reflect the contamination in cleaning, transportation, handling, storage, and marketing [18]. Thus, these results indicate the major sources of microbial contamination in tomato at western Nepal are the postharvest activities. The farmers generally use underground water for irrigation and washing which is usually considered as safe and clean. But use of unhygienic containers in transportation and storage may have increased the microbial contamination. The fresh produce (fruit and getable) can be contaminated with coliform group of bacteria through irrigation and washing water and manure. Therefore, to manage the coliform bacteria below detectable level the production practices such as use of safe and well-decomposed manure and clean water for irrigation and washing should be done. Similarly, methods of postharvest transportation, storage, and handling also seemed to be major contaminants in this study. Therefore, it seems necessary to adopt the improved postharvest handling practices by traders to maintain lower level of the microbial population. Here we found washing with clean tap water, grading, and transportation in clean plastic crates have positive impact in reducing the hazardous microbial load in tomato after harvest. Not only were the improved practices of transportation to be superior in reducing microbial population in tomato supply chain, but also it is important to reduce foodborne illness, to decrease spoilage, to enhance the shelf life, and to improve appearance and nutritive value [19].

### 3.3. Effect of Varieties in Microfloral Population in Tomato

Microfloral population and its composition can be differing according to the variety in tomato. We observed similar results in the present study. In this study, we tested four different tomato cultivars for the postharvest microfloral population. Here we observed the cultivar CLN 369A as the most supportive to all the microbes among the tested varieties. Significantly the highest log-transformed CFU per gram of aerobic, coliform, and yeast population was recorded in CLN369A (5.19, 5.10, and 3.51, resp.) except mold population which was highest in CLN 3940 (Table 3). While this microbe population was recorded to be lowest in Pusa ruby, the values were 2.58, 4.53, 0.96, and 1.77 log CFU per gm, respectively. Among the tested varieties, the aerobic acterial population was above the detectable level in CLN 369A and CLN 3940 while the coliform bacterial population was above detectable level in all the cultivars used in the study. The mold and yeast population were found to be lower than the detectable level in all the tested cultivars. Phenotypic variation in plant health and nutritional status in different varieties generally determine the colonization of microorganism in the surface [20].

**Figure 2:**
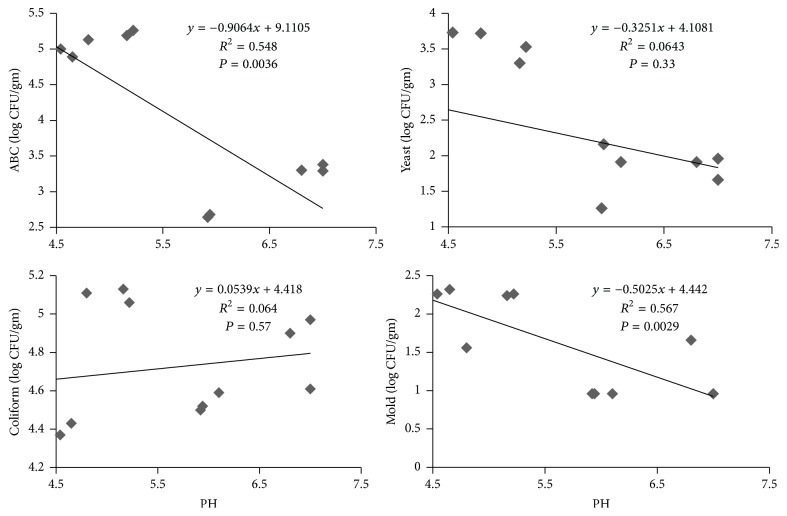
Correlation between the tomato fruit juice pH and microbial population. *R*^2^ value is adjusted value.

Moreover, the correlation between microbial load and pH was also analyzed. We found strong negative correlation between aerobic bacterial population versus pH and mold versus pH, while positive correlation was observed between coliform population and pH (Figure 2).

There are many reports on the differential response of tomato varieties with preharvest as well as postharvest pathogen colonization in tomato. Xia et al. [21] described the higher colonization of* Salmonella enterica* in certain tomato varieties but not in others. The postharvest microbial colonization is mainly associated with water uptake during submergence, acidity, porosity of stem scars, physical and chemical properties of the vascular bundles, and wounding and scratching in transportation and handling [21]. Similarly, Beuchat [3] classified intrinsic and extrinsic factors which are responsible for the population composition of microorganism colonizing in perishable produce. The extrinsic factors include the environment where produce has been grown and storages while intrinsic factors include nature of the epithelium and protective cuticle, pH, and the presence of antimicrobials in fruit pulp and tissues. Thus, the intrinsic factors are totally cultivar dependent while extrinsic factors are environment dependent. Therefore, use of appropriate variety is another important factor to be considered for the reduction of microbial hazards in fresh fruit and vegetable. Zepeda-Lopez and Gonzalez-Lugo [22] also obtained similar results. They concluded that the coliform bacteria could not grow in acidic condition. Dingman [23] also recorded increased population of* E. coli* (coliform bacteria) in apple with increased pH and reduced population with reduction in pH. There are many previous reports that the mold and yeast can grow in low pH condition, since they utilized the organic acid for their growth and development [3].

The results of the study showed the selection of proper variety could be useful to reduce the hazardous microbial population in perishable fruits and vegetables. The higher microbial load in tomato was found to be associated with lower level of pH.

From the present study it can be summarized that the microbial population is significantly differed according to the means of transportation, stage in supply chain, and varieties used. Thus, it can be concluded that there are many spaces to manage the microbial population in fresh product. If proper means of transportation and packaging procedures are followed with use of appropriate variety, the contamination of microbes can be reduced and ultimately have less effect on human health, and the shelf life of the perishable goods can be enhanced.

There is a need to make the public, farmers, and traders in particular aware of the risk involved in the use of waste contaminated water and untreated manure during production and after production. Prevention of vegetable contamination with microorganisms should be the responsibility of everyone involved in the preharvest, harvest, postharvest, transportation, and marketing operations to assure that fresh produce is safe for human consumption.

## Figures and Tables

**Table 1:** Difference between conventional and improved methods of transportation.

Conventional method (farmer's practice)	Improved practice
(i) No washing (ii) No grading (iii) Transportation in bamboo basket plastered with mud and dung	(i) Washing with tap water after harvesting (ii) Grading for overripe, normal, and underripe tomato fruit (iii) Transportation in clean plastic crate

**Table 2:** The microbial population affected by postharvest management in fresh tomato.

Practice	ABC (log⁡CFU g^−1^)	Coliform (log⁡CFU g^−1^)	Mold (log⁡CFU g^−1^)	Yeast (log⁡CFU g^−1^)
Conventional	4.77^a^	5.07^a^	2.35^b^	2.98^a^
Improved	4.65^a^	3.41^b^	2.59^a^	2.09^b^

*P* value	0.49	0.00	0.05	0.00
CV	20.35	17.76	15.55	23.77
LSD	0.56	0.44	0.22	0.35

Means followed by the same letter(s) do not differ significantly at 1% level of probability, *P* = probability value, CV = Coefficient of Variation, and LSD = Least Significant Difference.

**Table 3:** Microbial population in different tomato cultivars at RARS, Khajura.

Cultivars	ABC (log⁡CFU g^−1^)	Coliform (log⁡CFU g^−1^)	Mold (log⁡CFU g^−1^)	Yeast (log⁡CFU g^−1^)
CLN369A	5.19^a^	5.10^a^	2.02^a^	3.51^a^
CLN 3940	4.96^b^	4.42^c^	2.29^a^	1.98^ab^
UC204A	3.32^c^	4.82^b^	1.19^b^	1.84^b^
Pusa ruby	2.58^d^	4.53^c^	0.96^b^	1.77^b^

*P*-value	0.00	0.00	0.00	0.09
CV	2.21	2.13	16.40	34.55
LSD	0.18	0.20	0.53	1.57

Means followed by the same letter(s) do not differ significantly at 1% level of probability, *P* = probability value, CV = Coefficient of Variation, and LSD = Least Significant Difference.
